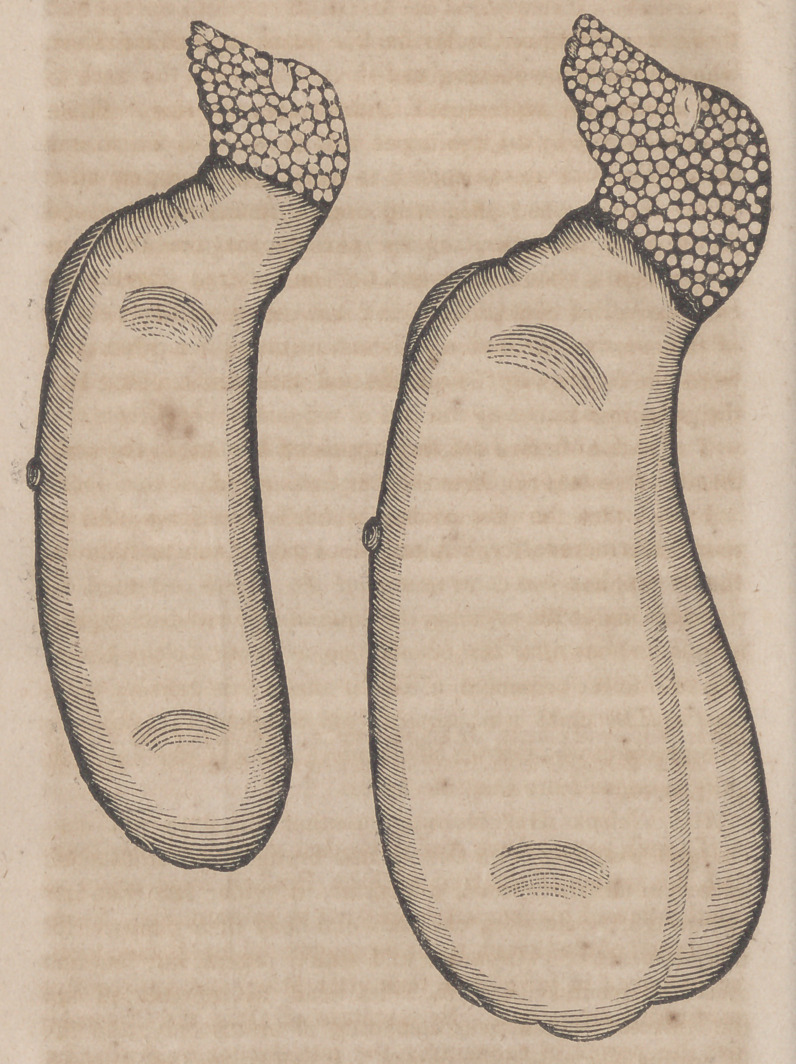# An Account of a Singular Case of Fœtal Monstrosity

**Published:** 1829

**Authors:** John Cook Bennett

**Affiliations:** McConnelsville, Ohio


ART. VI.—*An account of a singular case of Fœtal Monstrosity.* By Dr. JOHN COOK BENNETT, of McConnelsville, Ohio.

When residing in Circleville, the following extraordinary case fell under my observation:—

A lady, in the sixth week of utero-gestation, was frightened in the street by the fighting of two dogs, one of which was mad. This was in the month of April. Immediately after the event, she was seized with uterine hæmorrhage, which continued for 24 hours, and the same discharge returned, in a less degree, once a fortnight, till she suffered abortion in June. On the 30th of May, she was seized with a violent inflammation of the left eye, which returned for three successive days, and after an ineffectual resort to cupping and blistering, was cured by the loss of a quart of blood from the arm. On the 4th of June, the uterine hæmorrhage returned. On the 5th, I was requested to visit her, and found that abortion was likely to take place; as tolerably strong uterine pains recurred every few minutes. I gave opium and laudanum freely, but the contractions of the uterus increased in violence; and in the evening, the liquor amnii was discharged. In half an hour after this occurrence, or about 8 o'clock, the arm of a fœtus presented, and soon afterwards delivery took place. The child was perfect, and weighed 3 1-2 ounces. The placenta was thrown off about 11 o'clock, and weighed half an ounce more than the fœtus.

At 7 o'clock next morning, another placenta was discharged weighing 5 1-2 ounces, and brought with it, attached by an umbilical cord, a monster, of rather less than its own weight, resembling the body and head of a puppy. It was destitute of extremities and sexual organs, but had an anus and meatus urinarius. Its head, as represented in the annexed sketch, was composed of brains only, and in its outline was essentially canine. It had neither eyes, ears, nor mouth, but was marked with lines and spots indicating the situation of those organs. It was not subjected to dissection.

At 4 o'clock, on the same day, another monster, with its placenta, was thrown off. It weighed 6 1-2 ounces, and the placenta 6. It resembled the first in all respects, except that there was attached to its back a mass resembling liver, which extended, widening and thickening, from the neck to the sacrum, as represented in the larger drawing. When detached, it weighed one ounce, which was also the weight of the head. I was permitted to make a hasty examination of the thoracic and abdominal organs of this monster; and found them all natural, except perhaps that the liver was larger, than is common in a fœtus of such a size. It weighed two ounces. I could not obtain permission to preserve either of the monsters, or scarcely to examine them, so unpleasantly were the feelings of the patient and attendants, affected by the phenomenon.

The patient before delivery appeared like one in the sixth month of gestation. Her recovery was rapid.

I am aware that the account which I have given, will be read with incredulity, but am prepared to substantiate all that it contains.

*September*, 1829.

## Figures and Tables

**Figure f1:**